# Age-stratified analysis reveals arterial thrombosis as a predictor for gender-related second cancers in myeloproliferative neoplasms: a case-control study

**DOI:** 10.1038/s41408-024-01052-4

**Published:** 2024-04-22

**Authors:** Arianna Ghirardi, Alessandra Carobbio, Paola Guglielmelli, Alessandro Rambaldi, Valerio De Stefano, Alessandro M. Vannucchi, Ayalew Tefferi, Tiziano Barbui

**Affiliations:** 1 FROM, Fondazione per la Ricerca Ospedale di Bergamo ETS, Bergamo, Italy; 2 Dipartimento di Scienze Mediche e Chirurgiche, Materno-Infantili e dell’Adulto, Università di Modena-Reggio Emilia, Modena, Italy; 3 CRIMM, Azienda Ospedaliera Universitaria Careggi, Dipartimento di Medicina Sperimentale e Clinica, Università di Firenze, Florence, Italy; 4 Divisione di Ematologia, ASST Papa Giovanni XXIII, Bergamo, Italy; 5 Dipartimento di Oncologia ed Emato-Oncologia, Università degli Studi di Milano, Milan, Italy; 6 Institute of Hematology, Catholic University, Rome, Italy; 7 Fondazione Policlinico Universitario A. Gemelli Istituti di Ricovero e Cura a Carattere Scientifico (IRCCS), Rome, Italy; 8 Division of Hematology, Department of Internal Medicine, Mayo Clinic, Rochester, MN USA

**Keywords:** Risk factors, Myeloproliferative disease

**TO THE EDITOR:**

Over the past decade, observational and population-based studies have documented an excess of solid tumors and lymphomas in patients with myeloproliferative neoplasms (MPN), including polycythemia vera (PV), essential thrombocythemia (ET) and myelofibrosis (MF), but the causes of this excess remain elusive [1–3].

It is well known that unprovoked venous thromboembolism (VTE) may precede second cancer (SC), but the possibility that arterial thrombosis may signal impending malignancy has only recently been explored in the general population [4].

We recently investigated this association in a case-control study in the context of MPN [5, 6] and found that the frequency of arterial thrombosis in PV, ET, and MF was significantly higher in those who subsequently developed SC (6.2% vs. 3.7%; *p* = 0.015). On multivariable analysis, the odds ratio for arterial thrombosis was 1.97 (95% CI 1.14–3.41) [6].

To ensure comparability, the matching process between cases and controls included sex, age at MPN diagnosis, date of diagnosis, and duration of MPN disease, thus masking a possible influence of age on events and affecting the applicability of these findings across different age groups.

To investigate whether the association between arterial thrombosis and SC was age-related, in the current analysis cases and matched-controls were stratified into two groups <60 or ≥60 years of age at MPN diagnosis. The age cut-off of 60 years was chosen because it conventionally represents low and high-risk categories prone to thrombosis.

**Fig. 1:**
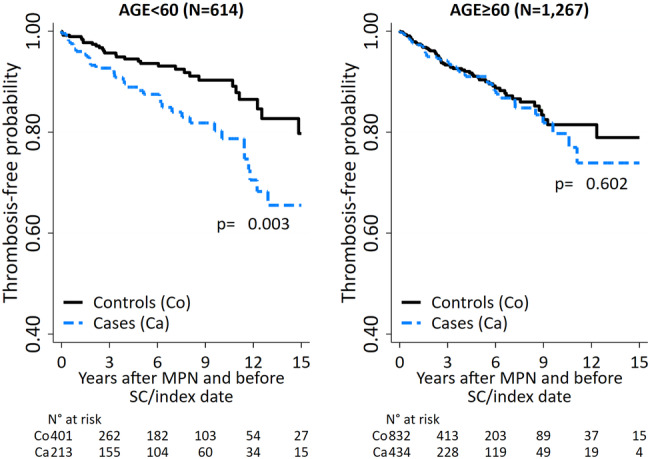
Thrombosis-free probability in cases and controls by age at MPN diagnosis. The figure on the left shows the thrombosis-free curves for cases and controls aged under 60 years, while the figure on the right shows the curves for cases and controls aged over 60 years.
(iii) In SC patients, the temporal distribution of total thrombosis in the years before cancer is shown in Fig. 2A. Interestingly, in younger patients, the proportion of total thrombosis before cancer diagnosis remained stable at around 25%, whereas in patients over 60 years of age, the frequency tended to increase progressively over the years, peaking at 41% in the two years before cancer. Notably, as described above, there was a significant difference in the type of cancer in the two groups with different patterns of thrombosis (Fig. 2B). According to the TNM classification, these second cancers were T1-2 N0 M0.

(i) Table 1S reports the characteristics of cases and controls in patients stratified by age under and over 60 years.
  MPN cases with a SC were 647 (PV: *N* = 216, ET: *N* *=* 317, MF: *N* = 114), and MPN matched-controls were 1234. In the under 60 group, the median age was 52 years (IQR 45–56), there was a slight preponderance of females in both cases and controls (53%), a lower percentage of ET patients in cases (50.2% vs. 58.6%, *p* = 0.047), a trend towards a higher frequency of patients with JAK2 mutations in cases compared to controls (74.2% vs. 67.3%, *p* = 0.079) as well as ASXL1 mutations (27.8% vs. 6.7%, *p* = 0.086). However, the latter information was only available for 48 patients out of a total of 614. The frequency of arterial and venous thrombosis was the same in cases and controls.
  No difference in cases and controls was observed in patients over 60, except for a higher frequency of leukocytosis in cases (34.6% vs. 28.1%, *p* = 0.017).
(ii) Table 2S provides details of events in these two groups. The most frequent category of SC was carcinoma seen in 141 (66.2%) and 285 (65.7%) patients with age under or over 60 years, respectively. The frequency of breast, uterus, ovary, and endocrine cancer was higher in younger (*p* = 0.001) whereas colorectal and lung cancers prevailed in over 60 (*p* = 0.001 and 0.045). The two groups showed no difference in the frequency of melanoma, non-melanoma skin cancer, and hematological malignancies.
  During a median follow-up of 3.6 years, cases and controls in both groups were exposed to the same cytoreductive drugs (Table 2S). The proportion of cases under 60 years treated with low-dose aspirin was lower than controls (74.6% vs. 84.3%, *p* = 0.004).
  Seventy thrombotic events (11.4%) were recorded in the group aged <60 years: 17 and 20 (in controls and cases, respectively) were arterial (4.2% vs. 9.4%, *p* = 0.011) and 75% involved the cerebral arteries. There was also a trend towards a higher frequency of venous thrombosis in cases compared to controls under 60 years (7.5% vs. 4.2%, *p* = 0.087). In contrast, thrombotic events were evenly distributed in cases and controls over 60 years of age (Table 2S).
  This different pattern is graphically illustrated in Fig. 1.

**Fig. 2:**
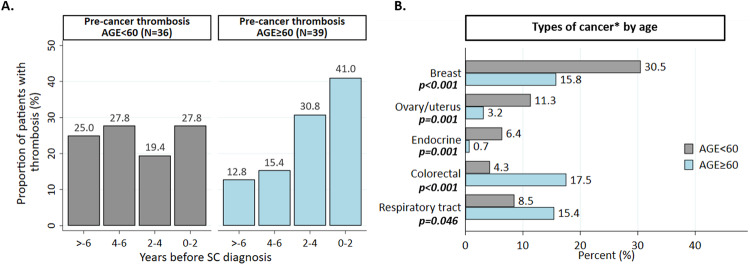
Dynamic of thrombosis before SC and type of malignancies. Dynamics of thrombosis in the years before cancer (**A**) and different cancer types (**B**) by age at MPN diagnosis. *No significant difference between the two groups for the other cancers (upper gastrointestinal tract, liver/pancreas, head&neck, prostate/urinary tract, kidney, cerebral, melanoma, non-melanoma skin cancer, and lymphoproliferative disease).
(iv) In the multivariable conditional logistic regression model (Fig. 1S) no predictors of second cancer emerged in patients over 60, except for a trend towards a 27% increased risk in patients with leukocytosis at the time of MPN diagnosis (OR = 1.27, *p* = 0.092). In younger individuals, the following factors independently influenced the risk of SC: (i) myelofibrosis phenotype compared to ET (OR 2.54, *p* = 0.056) (ii) arterial thrombosis, i.e., myocardial infarction, stroke (OR 2.53, *p* = 0.011) (iii) aspirin therapy, which reduced this risk (OR 0.52, *p* = 0.006).

This study has revealed age-related differences in the frequency of thrombosis in younger patients with MPNs before the onset of second cancers. In particular, arterial occlusion leading to cerebral transient ischemic attack, stroke or myocardial infarction emerged as an independent predictor of SC in association with the myelofibrosis phenotype. Interestingly, multivariable analysis also highlighted aspirin use as a protective factor against these cancers [7].

It is noteworthy that post-thrombotic SC were predominantly diagnosed in younger women and were more likely to include breast, uterine, and thyroid cancer compared to older individuals, where this group of SC was as common in cases as in controls. Conversely, in MPN patients over the age of 60, thrombotic events were as common in cases as in controls. Notably, all these cancers were localized, suggesting that regular monitoring of MPN may have allowed early detection of these malignancies. Notably, the frequency of thrombosis in the younger group was 25% every couple of years prior to cancer, whereas in cases over 60 years of age, it increased progressively and peaked in the 2 years prior to cancer diagnosis.

In the general population, this association has been well-documented in population-based studies. Following stroke, the cancer incidence rate increased by 20% compared to the age-adjusted rate [8], and similar results were observed after myocardial infarction and peripheral arterial thrombosis [9, 10]. There is a growing body of evidence elucidating the pathogenic mechanisms underlying this association. Chronic inflammation induced by trigger factors such as age-related clonal hematopoiesis of indeterminate potential (CHIP), smoking, alcohol consumption, obesity, and diabetes is now recognized as a major contributor to atherosclerosis, thrombosis, and cancer, ultimately leading to death [11]. In our analysis, no difference was found in the frequency of obesity, alcohol/smoking habits, and family history of cancer in cases and controls between both age groups.

In MPNs clonal hematopoiesis supported by driver and non-driver gene mutations may elevate the risk of premature atherosclerosis and compromise tumor immune surveillance [3]. The associated hyper-inflammation state, coupled with increased hematocrit, leukocytosis, thrombocytosis, endothelial cell activation, and hyper-coagulation, are acknowledged as pivotal elements in the pathogenesis and progression of MPNs, potentially influencing the development of solid tumors [3]. This is particularly evident for MF which, in our analysis, emerged as an independent risk factor for cancer likely due to blood monocytosis [12].

Our observations reveal a distinct temporal relationship between tumor onset and occurrences of stroke and myocardial infarction in two age-stratified groups. In the elderly group, there appears to be a potential contribution of colorectal and lung cancers to thrombosis likely explained by the thrombogenic properties inherent in these cancer cells [4]. Conversely, this effect appears less pronounced in female cancers affecting the breast, uterus, ovaries, and endocrine organs. In these cases, factors more closely linked to MPN-driven inflammation may be predominant.

This study has inherent limitations that warrant acknowledgment. Firstly, the findings are derived from a post-hoc analysis of a database initially designed to document second tumors in individuals with MPNs. Consequently, these results should be regarded as hypothesis-generating and necessitate validation through future confirmatory research. Secondly, there is a likelihood that patients experiencing fatal thrombosis were excluded, introducing a potential selection bias. This exclusionary criterion could impact the generalizability of our findings and should be taken into consideration in the interpretation of the results. Lastly, the constrained frequency of MPN diseases contributes to a relatively limited number of vascular events and tumors within the study cohort. This scarcity poses a challenge in achieving robust statistical power and warrants caution in extrapolating the findings to broader populations.

In conclusion, our findings provide a novel perspective on the predictive significance of new-onset arterial thrombosis in MPNs as a potential risk factor pathogenetically and temporally correlated with the onset of solid cancer. Further investigations are required to validate these novel findings and improve our understanding of the identification of biomarkers for monitoring MPN patients after stroke, myocardial infarction, and probably also after venous thrombosis.

### Supplementary information

Table 1S

Table 2S

Figure 1S
