# Supplementary material for: Age-stratified analysis reveals arterial thrombosis as a predictor for gender-related second cancers in myeloproliferative neoplasms: a case-control study
Source: Blood Cancer J. 2024 Apr 22;14(1):68. doi: 10.1038/s41408-024-01052-4 (PMC11035557; doi:10.1038/s41408-024-01052-4)
Supplement: Supplementary file 1 — Table 1S [file 41408_2024_1052_MOESM1_ESM.pdf]

Table 1S. Characteristics of cases/controls by age at MPN diagnosis

	AGE<60 (N=614)				AGE≥60 (N=1267)			
	N non missing	CONTROLS N=401	CASES N=213	p	N non missing	CONTROLS N=833	CASES N=434	p
**Age at MPN diagnosis**	614	52.0 (45.0-56.0)	52.0 (46.0-56.0)	mv	1,267	69.0 (65.0-74.0)	69.0 (65.0-74.0)	mv
**Male gender**	614	190 (47.4)	100 (46.9)	mv	1,267	435 (52.2)	231 (53.2)	mv
**Years from MPN to SC**	614	5.7 (2.0-9.6)	6.7 (3.4-10.9)	mv	1,267	3.2 (1.3-6.2)	3.5 (1.1-6.7)	mv
**PV**	614	111 (27.7)	71 (33.3)	0.14	1,267	283 (34.0)	145 (33.4)	0.84
**ET**	614	235 (58.6)	107 (50.2)	**0.047**	1,267	407 (48.9)	210 (48.4)	0.87
**MF (primary and secondary)**	614	55 (13.7)	35 (16.4)	0.37	1,267	143 (17.2)	79 (18.2)	0.65
**Symptomatic disease at diagnosis**	614	70 (17.5)	39 (18.3)	0.79	1,267	153 (18.4)	86 (19.8)	0.53
**Splenomegaly at diagnosis**	602	79 (20.1)	48 (23.0)	0.41	1,242	182 (22.2)	90 (21.2)	0.68
**Genetics & molecular data**								
JAK2 V617 mutation	614	270 (67.3)	158 (74.2)	*0.079*	1,267	630 (75.6)	330 (76.0)	0.87
≤50%	315	153 (78.9)	96 (79.3)	0.92	710	347 (74.0)	177 (73.4)	0.88
>50%	315	41 (21.1)	25 (20.7)			122 (26.0)	64 (26.6)	
EXON-12 mutation	614	2 (0.5)	2 (0.9)	0.52	1,267	5 (0.6)	4 (0.9)	0.52
CALR mutation	614	58 (14.5)	22 (10.3)	0.15	1,267	67 (8.0)	43 (9.9)	0.26
MPL mutation	614	15 (3.7)	4 (1.9)	0.20	1,267	22 (2.6)	9 (2.1)	0.54
**Non-driver mutations**	48	9 (30.0)	8 (44.4)	0.31	94	42 (64.6)	18 (62.1)	0.81
ASXL1	48	2 (6.7)	5 (27.8)	*0.086*	94	25 (38.5)	10 (34.5)	0.71
TET2	48	4 (13.3)	1 (5.6)	0.64	94	9 (13.8)	7 (24.1)	0.22
EZH1	48	0 (0.0)	0 (0.0)	-	94	1 (1.5)	0 (0.0)	1.00
**Abnormal karyotype**	353	15 (6.6)	5 (4.0)	0.33	651	45 (10.5)	26 (11.8)	0.61
**CV risk factors***	614	242 (60.3)	124 (58.2)	0.61	1,267	643 (77.2)	324 (74.7)	0.31
**Lab parameters at MPN diagnosis**								
Hb	614	14.7 (13.4-17.0)	14.9 (13.5-17.0)	0.30	1,267	14.9 (13.4-17.2)	15.0 (13.2-17.5)	0.47
HCT	614	43.1 (39.2-49.0)	43.2 (40.0-50.0)	0.37	1,267	45.0 (40.3-52.0)	45.3 (40.0-52.0)	0.84
WBC	614	9.0 (7.3-11.4)	9.2 (7.6-12.0)	0.17	1,267	9.2 (7.4-11.9)	9.7 (7.5-12.5)	*0.065*
>11 x10^9/L	614	107 (26.7)	67 (31.5)	0.21	1,267	234 (28.1)	150 (34.6)	**0.017**
PLT	614	656.0 (509.0-810.0)	623.0 (479.0-800.0)	0.11	1,267	609.0 (422.0-767.0)	622.0 (448.0-771.0)	0.55
**Events before MPN diagnosis**								
**Thrombosis**	614	71 (17.7)	38 (17.8)	0.97	1,267	191 (22.9)	90 (20.7)	0.37

Arterial	109	37 (52.1)	25 (65.8)	0.27	280	149 (78.4)	64 (71.1)	0.31
Splanchnic	109	16 (22.5)	8 (21.1)		280	4 (2.1)	4 (4.4)	
Venous	109	18 (25.4)	5 (13.2)		280	37 (19.5)	22 (24.4)	

*CV risk factors: smoking, hypertension, dyslipidemia, diabetes, alcohol, obesity
