# Supplementary material for: Age-stratified analysis reveals arterial thrombosis as a predictor for gender-related second cancers in myeloproliferative neoplasms: a case-control study
Source: Blood Cancer J. 2024 Apr 22;14(1):68. doi: 10.1038/s41408-024-01052-4 (PMC11035557; doi:10.1038/s41408-024-01052-4)
Supplement: Supplementary file 2 — Table 2S [file 41408_2024_1052_MOESM2_ESM.pdf]

Table 2S. Type of Second Cancer (SC), treatments and thrombotic events between MPN and cancer by age at MPN diagnosis

	AGE<60 (N=614)			AGE≥60 (N=1267)			p (type of SC: <60 vs. ≥60)
	CONTROLS N=401	CASES N=213	p	CONTROLS N=833	CASES N=434	p	
**Type of Second Cancer (SC)**							
**Melanoma**	-	15 (7.0)	-	-	17 (3.9)	-	*0.085*
**Non-melanoma skin K**	-	39 (18.3)	-	-	88 (20.3)	-	0.55
**Non-skin solid K**	-	141 (66.2)	-	-	285 (65.7)	-	0.89
Breast	-	43 (30.5)	-	-	45 (15.8)	-	**<0.001**
Ovary/uterus	-	16 (11.3)	-	-	9 (3.2)	-	**0.001**
Colorectal	-	6 (4.3)	-	-	50 (17.5)	-	**<0.001**
Upper gastrointestinal tract	-	8 (5.7)	-	-	14 (4.9)	-	0.74
Liver/pancreas	-	4 (2.8)	-	-	11 (3.9)	-	0.78
Respiratory tract	-	12 (8.5)	-	-	44 (15.4)	-	**0.046**
Head&neck	-	4 (2.8)	-	-	11 (3.9)	-	0.78
Prostate/urinary tract	-	36 (25.5)	-	-	85 (29.8)	-	0.36
Kidney	-	1 (0.7)	-	-	4 (1.4)	-	1.00
Endocrine*	-	9 (6.4)	-	-	2 (0.7)	-	**0.001**
Cerebral	-	1 (0.7)	-	-	2 (0.7)	-	1.00
Others	-	1 (0.7)	-	-	8 (2.8)	-	0.28
**Lymphoproliferative disease**	-	18 (8.5)	-	-	44 (10.1)	-	0.49
**Treatments**							
**Aspirin**	338 (84.3)	159 (74.6)	**0.004**	659 (79.2)	333 (76.7)	0.31	-
**Single drug exposure (first-line monotherapy)**							
Hydroxyurea	123 (30.7)	72 (33.8)	0.43	512 (61.5)	245 (56.5)	*0.084*	-
Anagrelide	7 (1.7)	3 (1.4)	1.00	1 (0.1)	0 (0.0)	1.00	-
Interferon	12 (3.0)	6 (2.8)	0.90	1 (0.1)	0 (0.0)	1.00	-
Pipobroman	4 (1.0)	1 (0.5)	0.66	12 (1.4)	15 (3.5)	**0.018**	-
Busulfan	1 (0.2)	0 (0.0)	1.00	4 (0.5)	4 (0.9)	0.46	-
Ruxolitinib	1 (0.2)	3 (1.4)	0.12	6 (0.7)	7 (1.6)	0.13	-
**Thrombotic events from MPN-cancer**							
**Thrombosis**	34 (8.5)	36 (16.9)	**0.002**	66 (7.9)	39 (9.0)	0.51	-
Arterial	17 (4.2)	20 (9.4)	**0.011**	29 (3.5)	20 (4.6)	0.32	-
Acute coronary syndrome	5 (29.4)	3 (15.0)	0.43	8 (27.6)	6 (30.0)	0.85	-

TIA	7 (41.2)	10 (50.0)	0.59	9 (31.0)	7 (35.0)	0.77	-
Stroke	1 (5.9)	5 (25.0)	0.19	9 (31.0)	4 (20.0)	0.52	-
Other	4 (23.5)	2 (10.0)	0.38	3 (10.3)	3 (15.0)	0.68	-
Venous/Splanchnic	17 (4.2)	16 (7.5)	*0.087*	36 (4.3)	19 (4.4)	0.96	-
DVT	8 (47.1)	8 (50.0)	0.87	23 (63.9)	12 (63.2)	0.96	-
Splanchnic vein thrombosis	4 (23.5)	6 (37.5)	0.46	1 (2.8)	3 (15.8)	0.11	-
Cerebral vein thrombosis	1 (5.9)	0 (0.0)	1.00	0 (0.0)	1 (5.3)	0.35	-
Other	4 (23.5)	2 (12.5)	0.66	12 (33.3)	3 (15.8)	0.21	-

*Of 11 patients with endocrine cancer, 9 were female and 2 male patients.
