# Supplementary figures and images for: Age-stratified analysis reveals arterial thrombosis as a predictor for gender-related second cancers in myeloproliferative neoplasms: a case-control study
Source: Blood Cancer J. 2024 Apr 22;14(1):68. doi: 10.1038/s41408-024-01052-4 (PMC11035557; doi:10.1038/s41408-024-01052-4)

Figure 1S. Predictors of second cancer by age at MPN diagnosis

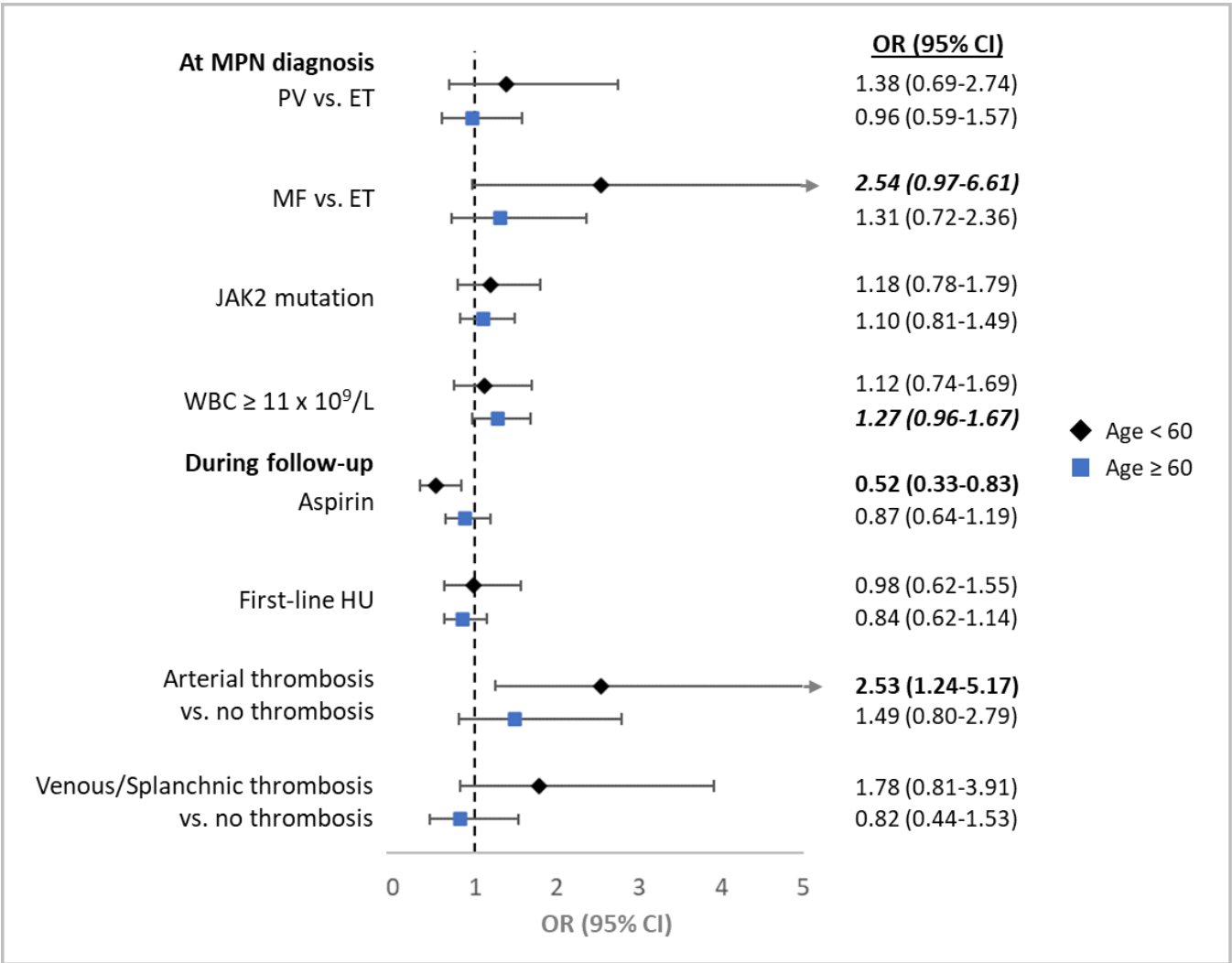

Supplement: Supplementary file 3 — Figure 1S [file 41408_2024_1052_MOESM3_ESM.pdf]
